# SMuRF: a novel tool to identify regulatory elements enriched for somatic point mutations

**DOI:** 10.1186/s12859-018-2501-y

**Published:** 2018-11-26

**Authors:** Paul Guilhamon, Mathieu Lupien

**Affiliations:** 1 Princess Margaret Cancer Centre, The MaRS Center, University Health Network, 101 College Street, Toronto, ON M5G 1L7 Canada; 2 Department of Medical Biophysics, University of Toronto, Toronto, ON Canada; 3 Ontario Institute for Cancer Research, Toronto, ON Canada

**Keywords:** Cis-regulatory elements, Mutations, Cancer, Enrichment, Transcriptional regulation

## Abstract

**Background:**

Single Nucleotide Variants (SNVs), including somatic point mutations and Single Nucleotide Polymorphisms (SNPs), in noncoding cis-regulatory elements (CREs) can affect gene regulation and lead to disease development. Several approaches have been developed to identify highly mutated regions, but these do not take into account the specific genomic context, and thus likelihood of mutation, of CREs.

**Results:**

Here, we present SMuRF (Significantly Mutated Region Finder), a user-friendly command-line tool to identify these significantly mutated regions from user-defined genomic intervals and SNVs. We demonstrate this using publicly available datasets in which SMuRF identifies 72 significantly mutated CREs in liver cancer, including known mutated gene promoters as well as previously unreported regions.

**Conclusions:**

SMuRF is a helpful tool to allow the simple identification of significantly mutated regulatory elements. It is open-source and freely available on GitHub (https://github.com/LupienLab/SMURF).

**Electronic supplementary material:**

The online version of this article (10.1186/s12859-018-2501-y) contains supplementary material, which is available to authorized users.

## Background

With the advent of next-generation sequencing technologies, a growing catalogue of genome-wide datasets has become available. This includes whole-genome sequencing to detect single nucleotide variants (SNVs) in diseased tissue (eg: TCGA Research Network: http://cancergenome.nih.gov/) as well as maps of histone variants and chromatin accessibility [1]. Using these datasets, numerous cis-regulatory elements (CREs) have been identified as recurrently mutated in cancer and other diseases. A notable example is the *TERT* promoter in glioma, melanoma, medulloblastoma, hepatocellular carcinoma, lung adenocarcinoma, thyroid and bladder cancers [2]. The mutations in this promoter create new transcription factor binding sites [3, 4], leading to increased *TERT* expression and ultimately immortalization and genomic instability [5]. Enhancers and anchors of chromatin interaction can also display recurrent mutation, such as the *PAX5* enhancer in chronic lymphocytic leukemia [6, 7] and CTCF binding sites in colorectal cancer [8].

Others have previously developed methods to identify important clusters of somatic point mutations based on proximity [9] or an enrichment compared to the local background [10]. However, the mutation rate of a CRE is impacted by its chromatin accessibility and the binding of transcription factors, as demonstrated by a lower rate of mutation in open compared to closed chromatin [11]. Therefore, recurrently mutated CREs should be identified against a background of other regulatory elements with a matched chromatin accessibility in the same cell or tissue type. To achieve this, SMuRF receives a user-defined set of regions of interest as the input rather than relying on a proximity clustering of SNVs and provides a user-friendly tool to identify, filter, and annotate significantly mutated genomic regions.

## Implementation

SMuRF consists of two main steps. The first filters, counts, annotates, and intersects the list of SNVs with the set of genomic coordinates, using a custom Bash script and the BEDTools suite [12]. The second consists in running a binomial test in R followed by a mutation rate filter to determine which genomic intervals are significantly enriched in SNVs and producing output figures as well as files for downstream analyses.

### Input processing

The SNVs in BED or vcf format, are optionally filtered for known SNPs. This will remove either all known SNPs or only those with a minor allele frequency above 1% to preserve potentially interesting acquired SNVs that also occur as extremely rare polymorphisms in the population.

Subsequently, the input genomic regions are annotated as either gene promoter regions or as distal regulatory elements. This is done by overlapping those genomic intervals with a catalogue of gene promoters, derived from Gencode transcription start site annotations [13].

Finally, the input SNVs and genomic intervals are intersected to map all SNVs to unique genomic intervals, and the resulting data structure forms the starting point of the statistical analysis for mutation enrichment.

All of the above filtering and annotating can be achieved with data from any genome for which the required annotation files are available. Those for human builds *hg19* and *hg38* are supplied with the tool for convenience.

### Identifying significantly mutated regions

The binomial test used by SMuRF to determine whether a given genomic region is significantly enriched for mutations requires an expected mutation rate. Depending on the sample cohort, the user can choose how this mutation rate is calculated. For each sample, the average number of mutations per base pair in input regions is calculated first. The “*allsamples”* option uses the average of those individual mutation rates across the entire sample cohort. However, if a subset of samples is more or less mutated than the rest, this could lead to biased results when a particular region contains mutations from that subset. For example, if a subset of samples is hypermutated relative to the rest of the cohort, this would artificially raise the background mutation rate, in effect reducing the number of significantly mutated elements identified. In these cases, the “*regionsamples”* option can be used, and the expected mutation rate when testing a particular region will be the average of the mutation rates for the individual samples mutated within that region only.

In both cases, the resulting *p*-value is then adjusted for multiple testing and the final set of regions is further filtered to include only those that pass a mutation rate threshold. This threshold is defined for each cohort by ranking the mutation rates for each region and identifying the inflection point, as previously described [14].

A number of output files are generated and these are detailed within the manual; they include a list of genes whose promoters are significantly mutated for use in gene ontology analyses, as well as a bed-formatted list of mutated regions annotated as distal regulatory elements to allow the user to associate them to target genes through GREAT [15] or C3D [16]. The main output figure is a scatter plot of -log10(q-value) against the number of unique samples mutated in the region, and color-coded to distinguish gene promoters from distal regulatory elements.

## Results and discussion

**Fig. 1:**
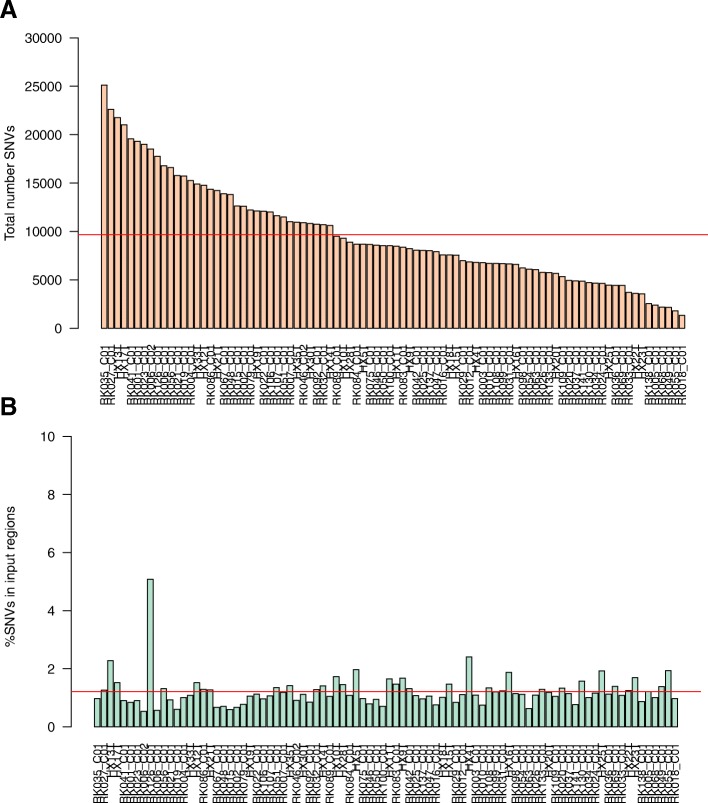
Overview of SNVs and their genomic distribution. **a**) The total number of SNVs in each sample considered in the analysis after filtering. They range from 1344 to 25,012. **b)** Percentage of SNVs falling within HepG2 open chromatin regions. Despite the range of total SNV numbers, the fraction that fall within the input genomic regions remains stable across the dataset, at 1.2% on average

**Fig. 2:**
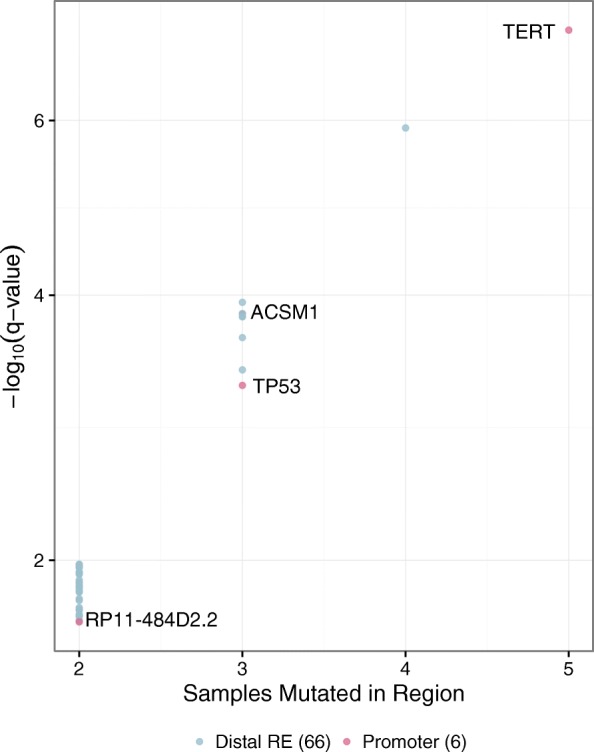
Significantly mutated regions identified by SMuRF. Each of the 72 genomic intervals that passed the significance (q-value ≤0.05) and mutation rate filters are represented. The negative log of the q-value calculated from the binomial test for each region is plotted against the number of unique samples with a mutation within that region. The most frequently and most significantly mutated regions include the promoters of both known and novel genes of interest in liver cancer

To illustrate the above steps, we used publicly available acquired SNVs from 88 liver cancer samples [17] and chromatin accessibility data from HepG2 [1] that provides a reference set for CREs. The total number of SNVs per sample used in the analysis after filtering ranged from 1344 to 25,121 (Fig. 1a), with an average of 1.2% falling within one of the 278,135 CREs (Fig. 1b) as identified in HepG2. While the input SNV numbers covered a wide range, no subset of patients was abnormally hyper or hypomutated, so we selected the “*allsamples”* mode to calculate the background mutation rate for each CRE. In total, 9485 individual CREs contained at least one mutation, of which 72 (6 promoters and 66 distal regulatory elements) were found to be significantly enriched for mutations (q-value ≤0.05 and peak mutation rate ≥ threshold) (Fig. 2 and Additional file 1: Table 1). These regulatory elements were each recurrently mutated in 2–5 samples.

Among the highly mutated promoters were those for the *TERT*, *TP53*, *ACSM1*, *TNFRSF8*, and *PCGF5* genes, all previously reported recurrently mutated regions in liver cancer [18]. Also significantly mutated, however, was the promoter of a gene with unknown function, *RP11-484D2.2*, highlighting the potential of this type of analysis for uncovering novel regions of interest.

**Fig. 3:**
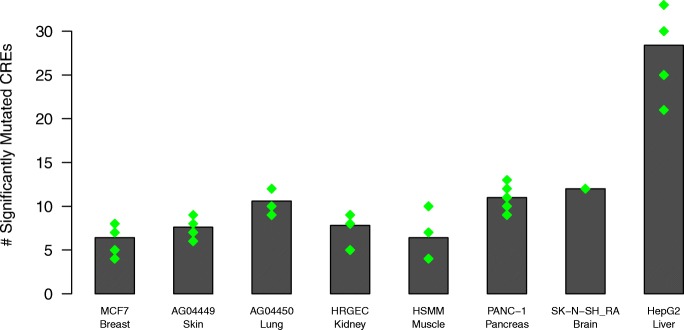
Assessing the sample specificity of SMuRF. SMuRF was run on matched chromatin accessibility data from seven other tissue types. Each peak set was randomly sampled 5 times and SMuRF was run on each iteration. SK-N-SH_RA had the lowest peak number and was not sampled. The selected peak sets were also matched to the HepG2 dataset for peak length. The number of significantly mutated CREs identified by SMuRF in each run are shown as green diamonds, with the height of the bar for each tissue corresponding to the average CRE number

To further assess the ability of this approach to identify mutated regulatory elements that are relevant to the samples of interest, we compared the number of significantly mutated CREs identified in HepG2 to those found in other tissue types when using the same liver cancer mutation data. Chromatin accessibility data from eight ENCODE cell lines [1], including HepG2, was randomly sampled five times, matching for peak number and peak length, and SMuRF was run on each iteration using the same settings detailed above (Fig. 3). Significantly fewer (Mann-Whiney U test *p*-value range: 0.007–0.012) mutated CREs were identified in each of the seven other cell lines compared to HepG2.

## Conclusions

Whole-genome sequencing and chromatin accessibility data sets in numerous normal and diseased tissues are becoming more commonly available. SMuRF aims to help further our understanding of the importance of non-coding elements in disease initiation and progression, by highlighting those regulatory elements most likely to have a functional importance due to their high burden of mutation.

## Additional file

Additional file 1: SMuRF output for the 72 significantly mutated CREs in liver cancer. (TXT 13 kb)

## Abbreviations

CRE: Cis-regulatory element
SNP: Single nucleotide polymorphism
SNV: Single nucleotide variant
